# Implementation facilitators and barriers of person and family-centred emergency care

**DOI:** 10.4102/hsag.v29i0.2789

**Published:** 2024-11-06

**Authors:** Mari-Louise Joubert, Neltjie C. van Wyk, Ronell Leech

**Affiliations:** 1 Department of Nursing Science, Faculty of Health Sciences, University of Pretoria, Pretoria, South Africa

**Keywords:** consolidated framework for implementation research, person- and family-centred care, implementation science, qualitative research, emergency department

## Abstract

**Background:**

At the time of the research, the nurses in the designated hospital’s emergency department did not implement person- and family-centred care to the detriment of patients and families. They were, however, eager to embark on the implementation of the recommendations of the Registered Nurses Association of Ontario for person- and family-centred care.

**Aim:**

This study therefore aimed to explore and describe the possible implementation facilitators and barriers prior to the use of the association’s recommendations.

**Setting:**

The study included eight nurses with different specialisation fields and more than 5 years of experience in an emergency department.

**Methods:**

During focus group interviews with nurse participants, the domains of the Consolidated Framework for Implementation Research were used to explore whether the recommendations of the Registered Nurses Association of Ontario could be used to structure person- and family-centred care in the emergency department of the designated hospital in the Mpumalanga province in South Africa. The framework guided the deductive data analysis.

**Results:**

The identified facilitators referred to a positive match between the recommendations and existing practice in the department. The barriers referred to the department’s fast-paced work environment in which a combination of emergency and primary care is delivered.

**Conclusion:**

One of the facilitators referred to the participants being used to ongoing training by and communication from management to support their adjustment to improvements. One of the barriers referred to the department’s fast-paced work environment.

**Contributions:**

The article contributes to practice improvement with a description of the use of frameworks to explore possible facilitators and barriers prior to endeavours to implement recommendations.

## Introduction

When a family member is ill or injured, the functioning of the whole family may be disturbed (Bouchoucha & Bloomer 2021:133; Lissoni et al. 2020:106). Although the ill or injured person’s healthcare needs are nurses’ priority, the family’s concerns need to be addressed as they are involved in the long-term care. Person- and family-centred care focusses on creating cooperative relationships among patients, family members and nurses to benefit care outcomes (Fernandez-Martinez et al. 2022:8).

In May 2015, the Registered Nurses Association of Ontario published clinical best practice guidelines for person- and family-centred care. The association is a professional association representing professional nurses and students in Ontario, Canada. The person- and family-centred guidelines have practice recommendations for assessment, planning, implementation and evaluation of care in a therapeutic, collaborative and empowering relationship with patients and family (RNAO 2015:84–86 and 23–40; Table 1).

**TABLE 1:** Practice recommendations of the person- and family-centred care best practice guidelines of the Registered Nurses Association of Ontario.

Practice recommendations
**Assessment** Establish a therapeutic relationship with the person using verbal and non-verbal communication strategies to build a genuine, trusting and respectful partnership. Build empowering relationship with the person to promote the person’s proactive and meaningful engagement as an active partner in their health care. Listen and seek insight into the whole person to gain an understanding of the meaning of health to the person and to learn their preferences for care. Document information obtained on the meaning and experience of health to the person using the person’s own words.
**Planning** Develop a plan of care in partnership with the person that is meaningful to the person within the context of their life. Engage with the person in a participatory model of decision-making, respecting the person’s right to choose the preferred interventions for their health.
**Implementation** Personalise the delivery of care and services to ensure care is not driven from the perspective of the healthcare provider and organisation, by collaborating with the person on elements of care, roles and responsibilities in the delivery of care and communication strategies.
**Evaluation** Obtain feedback from the person to determine the person’s satisfaction with care and whether the care delivered was person and family centred.

*Source*: Regisered-Nurses Association of Ontario, 2015, *Clinical best practice guideline: Person- and family-centred care*, RNAO: Editorially Independent

At the time of the research, the nurses in the designated hospital’s emergency department did not use person- and family-centred care but wished to implement the recommendations of the Registered Nurses Association of Ontario. According to implementation science, it is necessary first to identify possible facilitators and barriers before the implementation commences in order to manage it appropriately during the planning and execution phases (Glasgow et al. 2012:1275). This study thus aimed to explore and describe the possible implementation facilitators and barriers prior to the use of the person- and family-centred care recommendations of the Registered Nurses Association of Ontario in the designated hospital emergency department.

### Context of the research

The study was conducted in an emergency department of a 221 bed multidisciplinary hospital in the Mpumalanga province of South Africa that consisted of three resuscitations units, two high care units, a wound care unit, six consulting cubicles with beds and four consulting areas with chairs. Patients involved in motor vehicle accidents and industrial injuries in commercial, mining and farming industries were treated by two permanent doctors, five sessional doctors and permanent nursing staff (four emergency care qualified professional nurses, four general professional nurses, three enrolled nurses and two nursing assistants). The team worked shifts of 12 h, from 06:45 to 19:00 and 18:45 to 07:00. A shift was usually covered by one doctor, one to two professional nurses, one enrolled nurse and one nursing assistant. At the time of the research, the annual number of patients reflected that 458 adults were treated after trauma; 625 adults with medical or surgical conditions; 293 children with medical conditions; 265 adults after injuries on duty; 186 adults for follow-up assessments after injuries on duty and 68 adults for wound care.

## Method

The Consolidated Framework for Implementation Research was used in the exploration of the facilitators and barriers. It consists of five domains (Keith, Crosson & O’Malley 2017:2) of which one relates to the intervention characteristics. It includes constructs that focus on the stakeholder’s perceptions of the advantages of implementing the intervention. Another domain addresses the inner setting of the intervention and includes constructs that relate to the organisation while the outer setting refers to the external context of the organisation. The fourth domain pays attention to the characteristics of the individuals involved with constructs about them, such as their knowledge about the intervention. The fifth domain focusses on the implementation process. Constructs referring to the planning, executing and evaluation of the intervention are addressed (Keith et al. 2017:2). The framework was used to construct the interview guide and to deductively analyse the data.

A qualitative study in conjunction with an implementation science strategy was done through focus group interviews to understand the facilitators and barriers prior to the use of the person- and family-centred care recommendations of the Registered Nurses Association of Ontario in the designated hospital emergency department.

### Study population and sample

The study population consisted of all the potential nurse participants who have met the inclusion criteria (Polit & Beck 2017:249) that referred to: (1) being nurses employed full time in the emergency department of the designated hospital and (2) having been involved in emergency nursing for at least 5 years. Twenty nurses from various categories with years of experience in the designated hospital’s emergency department formed the study population. An invitation to participate in the research was sent out to them with information about the research captured in a participant information leaflet that they could study at their own time. Only eight female nurses accepted the invitation to partake in the research (Table 2), representing 40% of the study population. Some of the potential participants were ill or on vacation leave and could not take part in the research.

**TABLE 2:** Participants’ demographic information.

Participants	Professional qualification	Academic qualification	Years of experience in nursing
NP1	Professional nurse	Diploma in nursing with a degree in emergency nursing science with nursing education	25
NP2	Professional nurse	Diploma in nursing science with a degree in critical care nursing with nursing education	20
NP3	Nursing auxiliary	Certificate in nursing	10
NP4	Nursing auxiliary	Certificate in nursing	8
NP5	Professional nurse	Diploma in nursing science with a degree in emergency nursing and nursing education	25
NP6	Professional nurse	Diploma in nursing science with a degree in neonatology nursing	20
NP7	Professional nurse	Diploma in nursing science with a degree in critical care nursing and nursing education	15
NP8	Professional nurse	Diploma in nursing science with a degree in nursing education	25

*Source:* Joubert, M., 2023 ‘Exploring facilitators and barriers to implement person- and family-centred care in a private hospital emergency department’, Masters in Nursing Science dissertation, University of Pretoria

### Data collection

The researcher invited the sample of eight nurses to the focus groups interviews and explained to them what the roles of the facilitators of the interviews would be. Two rounds of semi-structured focus group interviews were done. In the first round, six of the eight participants were present who discussed the person- and family-centred guidelines according to the first three domains, and in the second round, all of the eight participants took part in the discussion of the guidelines according to the last two domains of the Consolidated Framework for Implementation Research. The focus group interviews did not exceed 90 min each and were conducted with a guide based on the domains of the Consolidated Framework for Implementation Research. Each recommendation of the practice guidelines for person- and family-centred care of the Registered Nurses Association of Ontario was discussed according to the five domains. The dates and times of the interviews were pre-arranged, and two sessions were held where participants could be in person or join through the Microsoft Teams application. Participants gave permission for audio recordings to prevent information to be lost during interviews. The first and second authors facilitated the interviews and compiled the field notes.

### Data analysis

Deductive data analysis according to the domains of Consolidated Framework for Implementation Research was done (Breimaier et al. 2015:4). The first author coded the data and identified categories while the second author repeated the process and compared the categories that she identified with that of the first author. The final set of categories reflects the outcome of a discussion to reach consensus by the two authors.

### Trustworthiness of the findings

Achieving knowledge in implementation science requires methodological trustworthiness. A detailed description of the data collection and analysis processes was provided as the researchers strived to prevent biases and to obtain credibility of the findings. The reader of the report will get a good picture of how the data have been collected and analysed. The use of a guide for the data collection and a framework for the data analysis based on the constructs of the Consolidated Framework for Implementation Research enhanced the credibility of the findings (Tappen 2016:174). Focus group interviews and field notes were used in the data collection. A coder and co-coder analysed the data. The researchers provided a rich description of the data collection and analysis processes to enable readers to understand precisely how the research was done. They thereby provided an audit trail to contribute to the consistency of the findings (Tappen 2016:180). The transferability of the findings was enhanced with a comprehensive description of the context of the study and the demographic information of the participants (Tappen 2016:179).

### Ethical consideration

Ethical approval of the research proposal was obtained from the University of Pretoria Faculty of Health Sciences Research Ethics Committee (110/2020), and consent was obtained from the designated hospital’s research ethics committee (02122020/1). The participants provided written informed consent to take part in the research. Confidentiality of the data shared with the researcher was maintained.

## Results

The results indicated the factors that may serve as facilitators and barriers to the implementation of the person- and family-centred care recommendations. The following categories apply: recommendations match current practice and guideline to enhance current practice (intervention characteristics domain), obliged to render primary care, referrals and transfers (outer setting domain), teamwork within the department, supportive leadership (inner setting domain), manage resistance to change (characteristics of individuals domain), training for implementation and communication for implementation (process of implementation domain; Table 3).

**TABLE 3:** Domains and categories.

Domains	Categories
Intervention characteristics	Recommendations match current practice Recommendations to enhance current practice
Outer setting	Obliged to render primary care Referrals and transfers
Inner setting	Teamwork within department Fast-paced department Traumatised patients are treated Supportive leadership
Characteristics of individuals	Manage resistance to change
Process of implementation	Training for implementation Communication for implementation

*Source*: Joubert, M., 2023 ‘Exploring facilitators and barriers to implement person- and family-centred care in a private hospital emergency department’, Masters in Nursing Science dissertation, University of Pretoria

### Domain: Intervention characteristics

The constructs of the intervention characteristics in the Consolidated Framework for Implementation Research refer to the belief that the use of the recommendations will benefit the organisation and the degree of the complexity of the recommendations.

#### Category: Recommendations match current practice

The existing hospital policies had much in common with the person- and family-centred care recommendations of the Registered Nurses Association of Ontario and served as an implementation facilitator. As the planned changes are in line with existing policies, fewer barriers are foreseen with its implementation. The participants already focussed on delivering individualised care to patients, and they tried to involve the family members in the planning and execution of the care. Unfortunately, acute care is delivered in the department, and life-saving procedures are more important than the involvement of family members in decision making. It is, however, possible to focus on the latter after the patients’ conditions have been stabilised:

‘And in that way, you try by all means to comfort …’ (NP3 female, nursing auxiliary, 10 years nursing experience)

People, whether being injured or accompanying family members, experience anxiety and stress when they visit emergency departments. Life-threatening situations are associated with these departments, and nurses have little time to attend to their distress. It is a fast-paced and unpredictable environment, and the participants had to deal with multiple stressors while still caring for patients and family members. The participants were, however, very positive about the implementation of the recommendations and came up with innovative ways to execute it:

‘… [*C*]lient liaison between the doctor and patients waiting, and he works from 9 to 9. And it is kind of like a client liaison, or he doesn’t do any nursing work. He just goes from patient to patient. If the communication between the patient and family sitting in the waiting area, he builds the relationship.’ (NP6 female, professional nurse, 20 years nursing experience)

#### Category: Recommendations to enhance current practice

The data revealed that the implementation of the person- and family-centred care recommendations could enhance the practice in the emergency department. The participants considered the implementation of the recommendations as a means to ensure that trusting relationships between them, their patients and the family members are strengthened:

‘I believe that we can build a good, trusted relationship in our unit.’ (NP4 female, nursing auxiliary, 8 years nursing experience)

The participants, however, also acknowledged that the overwhelming number of patients that they had to manage could be a barrier to the implementation of the recommendations:

‘… [*W*]e have a big emergency department … it’s extremely busy. And to have a personal relationship with those patients is a big challenge in the unit for the staff.’ (NP6 female, professional nurse, 20 years nursing experience)

Securing patient privacy and confidentiality is essential in person- and family-centred care. In the designated department, the treatment bays were unfortunately separated by curtains only. Therefore, the outlay of the department served as an implementation barrier:

‘In the unit, we have private cubicles, but only divided by a curtain. So, you don’t get to meet them unless it’s in the results where you have to when the patient is normally you are able to have a one-on-one talk with your patient or after the doctor has seen them.’ (NP4 female, nursing auxiliary, 8 years nursing experience)

Effective communication in emergency departments empowers family members to understand why and how patients’ triaging is done and why some patients have to wait longer than others for treatment. The implementation of person- and family-centred care recommendations may, according to the participants, improve communication with family members:

‘… [*I*]t will help tell the family members in advance what’s going on and also let them know what’s going on with the persons in the unit. They will also be satisfied with the quality of service.’ (NP1 female, professional nurse, 25 years nursing experience)

### Domain: Outer setting

The constructs of the outer setting in the Consolidated Framework for Implementation Research refer to patients’ expectations and policies that may influence the implementation of innovation.

#### Category: Obliged to render primary care

Emergency departments are often misused by people with minor ailments as they wrongly view the departments as consulting rooms of general practitioners and become very upset when they have to wait until patients with life-threatening conditions have been stabilised. The misuse of the designated department contributed to the large number of patients treated in the department. It may according to the participants be considered as a barrier to the implementation of person- and family-centred care:

‘Usually when kids go to sports events, the parents sign consent saying that the children may be taken to the nearest facility for treatment … over weekends the emergency units are very busy ….’ (NP8 female, professional nurse, 25 years nursing experience)

At the designated department, people often require treatment for chronic conditions such as hypertension. It happened, according to the participants, especially over weekends and after hours when general practitioners’ consultation rooms and primary healthcare clinics are closed. Patient overloads are experienced that leave the staff with very little time to render person- and family-centred care to patients who need emergency care:

‘They will come during the night or on the weekends when there is really a staff shortage, and there are critically ill patients in the unit.’ (NP1 female, professional nurse, 25 years nursing experience)

‘They have got a headache over the weekend, and now they did not drink their blood pressure tablet for one day, so they go to the emergency department for it, where they will be seen.’ (NP8 female, professional nurse, 25 years nursing experience)

According to the participants, the designated emergency department is obliged to render care to casualties of neighbour mining industries. Some people who work for the mining industries in the area do not make use of occupational healthcare services but rather consult the emergency department:

‘We are in a mining community. Therefore, there are a lot of occupational health clinics in our midst …’ (NP8 female, professional nurse, 25 years nursing experience)

‘… [*W*]e got the very wide community that we have to serve.’ (NP1 female, professional nurse, 25 years nursing experience)

#### Category: Referrals and transfers

The participants recommended that person- and family-centred care should be implemented in the whole hospital and not only in one department. Patients get referred to and transferred from the emergency department to other departments, and the staff of all departments should be knowledgeable and skilled to continue with person- and family-centred care. The participants applauded the existing good communication between the departments that, according to their opinion, could serve as a facilitator for the implementation of person- and family-centred care:

‘… [*I*]f you are on shift and you know this person, and you were concerned about the patients, you follow up and see if the patient is okay and fine and if the patient will be fine when they go home. And you can follow up with the nurses in the ward so that they can also follow up with the patient if there was a concern.’ (NP2 female, professional nurse, 20 years nursing experience)

The referral of patients to other facilities is time consuming as complex processes have to be followed causing nurses to spend too much time on arranging transfers to the detriment of the delivery of person- and family-centred emergency care where required:

‘[*D*]ifficult to find placement for patients, but we cannot withhold treatment just because we know that we’re going to have a problem ….’ (NP7 female, professional nurse, 15 years nursing experience)

### Domain: Inner setting

The inner setting of the Consolidated Framework for Implementation Research refers to the organisation’s structure and communication channels, as well as its readiness to implement changes.

#### Category: Teamwork within department

Efficient teamwork is required to implement change, such as the implementation of the person- and family-centred guidelines of the Registered Nurses Association of Ontario to improve the quality of care in the emergency department of the designated hospital. The participants experienced cohesion within the team and expressed their satisfaction with team communication:

‘… [*T*]he group cohesion was very good, we worked together and helped each other more. I think it was much better than before we work together and then talk to each other ….’ (NP8 female, professional nurse, 25 years nursing experience)

The group cohesion that the participants experienced could be used as a facilitator for the implementation of the recommendations. Fortunately, the participants also experienced the implementation of change in the recent past as non-disruptive:

‘And also, there was the incorporate the whole lot of new staff quickly into the unit … we had to do very quick orientation and very quick group cohesion developed in order for things to be carried out.’ (NP7 female, professional nurse, 15 years nursing experience)

The participants were satisfied with the manner in which the nursing managers implemented hospital policies. They appreciated the opportunities to make recommendations regarding the implementation of the policies:

‘You get the policies, and then the nursing manager discussed it with us, and we discussed it, and if we did not agree, we could send it back to be changed as some proposed policies were not practical.’ (NP1 female, professional nurse, 25 years nursing experience)

#### Category: Fast-paced department

Emergency departments are considered as fast-paced units because of the life-saving care that is rendered. Nurses use a triage system and respond to patients’ need accordingly. Patients who do not need life-saving interventions are obliged to wait for treatment until patients who do require such interventions have been treated. Little time is available for nurses to develop trusting relationships with patients and their family members:

‘Actually, to make our patients feel welcomed and everything in the unit, because in most cases casualties are busy. So, our patient is not going to get one-on-one private session with the nurses compared to the wards. So, building this or implementing this (the PFCC recommendations) would actually create a very good therapeutic environment for our patients.’ (NP3 female, nursing auxiliary, 10 years nursing experience)

The fast-paced workflow in the emergency department of the designated hospital is a barrier to the implementation of the recommendations as there sometimes is not time to do a comprehensive assessment and to render care accordingly. Shortages of nurses aggravate the situation:

‘One’s sympathy levels are low, and every person that comes in doesn’t feel like an individual anymore.’ (NP6 female, professional nurse, 20 years nursing experience)

‘We tend to look unfriendly because even in your in your mind, you are triaging the patients to see who need emergency care.’ (NP7 female, professional nurse, 15 years nursing experience)

#### Category: Traumatised patients are treated

Patients visiting emergency departments often are emotionally distressed and feel traumatised because of the need to be treated for a serious illness or injury. The accompanying family members experienced similar emotions. They may believe that their loved ones should be treated as a matter of urgency while all other families share the same expectations:

‘We see here in our emergency department often people who are very emotional when they come in. It’s an emergency unit, but it doesn’t mean that turnaround time was forced or quick and that they were already stressed because they’ve got a family member not feeling well.’ (NP5 female, professional nurse, 25 years nursing experience)

Emergency departments are often experienced as stressful, but it should not be a barrier to the implementation of person- and family-centred care.

#### Category: Supportive leadership

Cultivating teamwork in fast-paced emergency departments is a daunting task for managers. They are required to support the nurses to attend to emergency situations and to patients who misuse the department for primary care purposes. Many in-service training opportunities encouraged the participants to improve their knowledge and skills:

‘Lots of in-service training in the emergency department. We are doing in-service training on specific topics where there really is a need for.’ (NP1 female, professional nurse, 25 years nursing experience)

The implementation of the recommendations will require a series of in-service training of all the nurses of the designated department. Fortunately, they are used to attending such sessions.

Utilising available tools to enhance interpersonal communication in the department may contribute to the implementation of the recommendations to foster person- and family-centred care:

‘… [*S*]ometimes you don’t feel like you can communicate exactly the problem … then the managers…come and sit in the meeting and to discuss the issues, and they facilitate communication so that it does not come off as punitive.’ (NP7 female, professional nurse, 15 years experience)

Effective communication is important for the implementation of improvement such as the person- and family-centred care recommendations.

### Domain: Characteristics of individuals

The characteristics of the individuals who will be involved in the implementation of innovation refer to their knowledge and beliefs about the intervention.

#### Category: Manage resistance to change

While some people enjoy changes at the work place, others do not. Managers who plan to implement changes should be prepared to manage staff who resist the implementation of change. If it is not done, the implementation will not be successful. The participants agreed that they tend first to resist changes, but as soon as they understand the necessity of the proposed changes, they usually cooperate:

‘We always resist at first because we have to ask why (is the change necessary)? We are the relay between the upper management and then the workforce. So, we need to know all the why’s, because the why’s are going to be directed at us at the end of the day, and we need to actually know what we are talking about to have changes made.’ (NP7 female, professional nurse, 15 years nursing experience)

The participants emphasised the importance of good communication to help the nurses understand why changes are important and necessary to ensure quality patient care. Changes should never be implemented in an autocratic manner:

‘So, sometimes you don’t feel like you can be blunt and communicate exactly the problem.’ (NP8 female, professional nurse, 25 years nursing experience)

The participants’ past experiences with the management of change may serve as a facilitator for the implementation of the person- and family-centred care recommendations. The participants emphasised the importance of enabling guidance from managers:

‘… [*S*]ome of us are still old school … when they introduce new things, it is when we think, no they think that they are better than us and then we become negative.’ (NP2 female, professional nurse, 20 years nursing experience)

### Domain: Process of implementation

The process of implementation refers to the planning of the implementation, engagement of participants and the execution of the innovation.

#### Category: Training for implementation

The participants described a need to be trained in implementing the person- and family-centred care recommendations in the designated department. They were used to attending workshops and conferences to improve their knowledge and skills in emergency care but have not been involved in change management. They were eager to learn new skills:

‘I think the training would actually help a lot.’ (NP3 female, nursing auxiliary, 10 years nursing experience)

The participants’ eagerness to improve their skills could serve as a facilitator for the implementation of the recommendations. According to the participants, the training of nurses to gain knowledge and skills to implement the recommendations will not on its own be sufficient. They should also be involved in each step of the planning for the implementation of the recommendations:

‘[*T*]he nurses must have the satisfaction that they are forming part of the guideline, to enhance the family centred care, the policy implementation, that sense of feeling that I have the skill and knowledge of how to be implanted, so that would be an incentive to me rather than getting a certificate.’ (NP7 female, professional nurse, 15 years nursing experience)

#### Category: Communication for implementation

Meetings to convey messages verbally, written notes to remind the stakeholders about strategies and posters with descriptions of the steps of implementation should be used to support the implementation of the recommendations. The participants agreed that ongoing communication would be needed to ensure a successful implementation of the recommendations. Fortunately, they have had experience in implementing changes in nursing procedures during the coronavirus disease 2019 (COVID-19) pandemic that might serve as a facilitator for the implementation of person- and family-centred care:

‘So, it was a whole rerouting of a lot of policies and procedures and we also had to do how a whole workflow change on every aspect … So, it was a big, big adjustment for the staff. We even had to do daily trainings for new work procedures every day, almost because things were changing so rapidly, especially in the first wave (of COVID-19) when it was still unknown.’ (NP7 female, professional nurse, 15 years nursing experience)

## Discussion

After discharge from care facilities, ill and injured persons are dependent on family members for care and support. Whether institutions officially use the person- and family-centred care guidelines of the Registered Nurses Association of Ontario or not, all nurses are obliged to prepare patients to take care of themselves with the assistance of family members after discharge (Malepe, Havenga & Mabusela 2022:9). A facilitator for the implementation of the recommendations in the designated emergency department was their existing policy to involve family members as much as possible in the planning and execution of care. Quality patient care is associated with a focus on the individual needs of patients and their relationships with loved ones. In emergency departments, nurses try to keep family members updated about the care that patients receive whether it is life-saving measures or primary care (Kuipers, Cramm & Nieboer 2019:19). By implementing the recommendations of the guidelines of the Registered Nurses Association of Ontario, the staff in the designated department may succeed to view the person as not only another patient that requires emergency care but as a whole person with unique family relationships (Emmamally, Erlingsson & Brysiewicz 2020:40). It will require from them to empower family members regarding continuation of care after discharge from the emergency department (Østervang, Lassen & Jensen 2022:7).

When life-saving procedures are performed, the injured or ill persons’ identity and personal needs tend to be neglected until their conditions stabilised. According to the participants, it may also happen that nurses’ focus remains on life-saving responsibilities to the extent that they neglect patients’ personal and interpersonal needs once they have recovered. This attitude may hinder the implementation of person- and family-centred care recommendations. According to Almaze and De Beer (2017:59), people who get admitted to emergency departments are stressed about their chances to recover from severe illnesses and injuries and so are their family members. They desperately need support from nurses to process their concerns (Byrne, Baldwin & Harvey 2020:8; Greenway, Butt & Walthall 2019:5–6). The triaging system that is used to indicate the severity of persons’ illnesses and injuries (Grover, Porter & Morphet 2017:93) should not replace their identity. All patients deserve to be treated as unique individuals with personal and interpersonal needs (Tam, Chung & Lou 2018:1).

The transfer of patients to other healthcare facilities is often challenging, leaving the staff of the emergency department to function as a general ward rather than a department where immediate care is rendered. According to Gorodetzer et al. (2020:6), emergency departments get overcrowded, and the healthcare teams are overwhelmed because of the reluctance of staff to admit patients who have been discharged from emergency departments to their wards and units. The participants identified challenges to refer and transfer patients for continuation of care and agreed that it might hinder the implementation of person- and family-centred care. Fortunately, the participants also identified that supportive leadership in their department contributed to their ability to manage the care of high numbers of patients. It may also enable the implementation of person- and family-centred care. Onboarding training programmes may help nurses to manage work-related stress effectively, improve their nursing skills (Glanz et al. 2018:353) and prepare them to implement innovation in emergency care (Senabye 2018:40–41). It may also build cohesion in nursing teams to collaboratively manage work-related demands (Buljac-Samardzic, Doekhie & Van Wijngaarden 2020:11).

According to the participants, the physical layout of the designated emergency department was not conducive to maintaining the privacy of patients. The cubicles were divided with curtains, and communication with one patient could be overheard by other patients. Such circumstances jeopardise opportunities to maintain trusting relationships with patients and their family members (Hartigan et al. 2018:2) and the implementation of person- and family-centred care guidelines. Poor layout of emergency departments and the management of large numbers of patients may abuse patients’ privacy rights. It leads to patient neglect, as nurses are obliged to keep all information of their patients confidential (De Steenwinkel et al. 2022:4). Such situations may also prevent patients and family members from expressing their healthcare needs (Al-Kalaldeh, Amro & Qtait 2020:29–35). That is a pity as family members should be encouraged to discuss their anxiety and concerns about their loved ones’ conditions with the nurses (Kwame & Petruckwa 2021:8).

Thorough planning for innovation may contribute to the successful implementation of the person- and family-centred care recommendations. The participants applauded their managers’ empowering leadership style and opportunities to be involved in the planning of innovation. The successful implementation of measures to improve and maintain quality patient care should always be a shared responsibility of the healthcare team (Stefánsdóttir et al. 2022:447). To implement and maintain quality person- and family-centred care in the designated emergency department, adequate resources and optimal professional development opportunities are non-negotiable.

### Implications and recommendations for practice

The implementation of the recommendations of the guidelines of the Registered Nursing Association of Ontario (RNAO) in the emergency department of the designated hospital is recommended to guide and assist in the improvement of the quality of care to the patients’ and family members’ satisfaction and to ensure desirable healthcare outcomes for all patients.

### Study limitations

Only 8 nurse participants of the study population of 20 took part in the research. The refusal of 12 potential participants to partake could be considered as a limitation of the study.

## Conclusion

The implementation facilitators and barriers of person- and family-centred care recommendations in the emergency department of the designated hospital were explored and described. The identified facilitators referred to a positive match between recommendations and existing practice in the department. The participants were used to ongoing training by and communication from management to support their adjustment to improvements. The barriers referred to the department’s high-paced work environment in which a combination of emergency and primary care is delivered.
